# Development of Low-Cost Inverted Microscope to Detect Early Growth of *Mycobacterium tuberculosis* in MODS Culture

**DOI:** 10.1371/journal.pone.0009577

**Published:** 2010-03-23

**Authors:** Mirko Zimic, Abner Velazco, Germán Comina, Jorge Coronel, Patricia Fuentes, Carmen G. Luna, Patricia Sheen, Robert H. Gilman, David A. J. Moore

**Affiliations:** 1 Unidad de Bioinformática y Laboratorio de Enfermedades Infecciosas, Laboratorios de Investigación y Desarrollo, Facultad de Ciencias y Filosofía, Universidad Peruana Cayetano Heredia, San Martín de Porras, Lima, Perú; 2 Laboratorio de Películas Delgadas, Facultad de Ciencias, Universidad Nacional de Ingeniería, Rimac, Lima, Perú; 3 Department of International Health, Johns Hopkins Bloomberg School of Public Health, Baltimore, Maryland, United States of America; 4 Department of Infectious Diseases and Immunity, Faculty of Medicine, Wellcome Centre for Clinical Tropical Medicine, Imperial College London, London, United Kingdom; 5 Clinical Research Unit, Department of Infectious and Tropical Diseases, London School of Hygiene and Tropical Medicine, London, United Kingdom; McGill University, Canada

## Abstract

**Background:**

The microscopic observation drug susceptibility (MODS) assay for rapid, low-cost detection of tuberculosis and multidrug resistant tuberculosis depends upon visualization of the characteristic cording colonies of *Mycobacterium tuberculosis* in liquid media. This has conventionally required an inverted light microscope in order to inspect the MODS culture plates from below. Few tuberculosis laboratories have this item and the capital cost of $5,000 for a high-end microscope could be a significant obstacle to MODS roll-out.

**Methodology:**

We hypothesized that the precise definition provided by costly high-specification inverted light microscopes might not be necessary for pattern recognition.

**Significance:**

In this work we describe the development of a low-cost artesenal inverted microscope that can operate in both a standard or digital mode to effectively replace the expensive commercial inverted light microscope, and an integrated system that could permit a local and remote diagnosis of tuberculosis.

## Introduction

The novel microscopic observation drug susceptibility (MODS) assay has been shown to be a sensitive, rapid and inexpensive method for the detection of the early growth of *Mycobacterium tuberculosis* (MTB) in liquid medium [1], [2], [3], [4], [5], [6], [7]. The MODS assay utilizes an inverted light microscope to visualize the characteristic cording growth of MTB through the underside of 24-well tissue culture plates previously inoculated with a decontaminated sputum sample and liquid culture media. This permits rapid, accurate detection of tuberculosis (TB) and multidrug resistant (MDR) TB at a unit cost of less than $3 per sample making it a potentially highly useful tool for resource-poor, high TB-burden countries [3]. MODS delivers direct drug susceptibility testing (DST) straight from the inoculated sputum sample which averts the need for manipulation of concentrated suspensions of *M tuberculosis* as is required for conventional direct DST–this reduces both cost and biohazard and the reduced number of procedural steps makes MODS an attractive option for decentralization to district laboratories. An inverted microscope (observing from beneath) is used to overcome two problems encountered by conventional microscopic visualization from above: (1) the distance from the objective to the bottom of the wells, where MTB colonies tend to aggregate, is greater than the working distance of a standard light microscope objective, and this difference increases if more magnification and numerical aperture (NA) is required, (2) condensation on the lid of the plate and obstruction and interference by the culture medium supernatant, often obscures the view from above.

However, an inverted light microscope is not a routine laboratory tool and the need to acquire one is a potential obstacle to MODS scale-up in developing countries. The average cost of $5,000 for a high-end model is particularly prohibitive because there are few other uses for the inverted microscope in the routine service laboratory.

We hypothesized that the precise definition provided by the high-specification inverted light microscope might not be necessary for the pattern recognition integral to the MODS assay. Here we describe the design and development of a low-cost device that could effectively replace the inverted light microscope and that could be integrated into a system to permit both a local and remote diagnosis.

## Materials and Methods

### Existing standard

**Figure 1:**
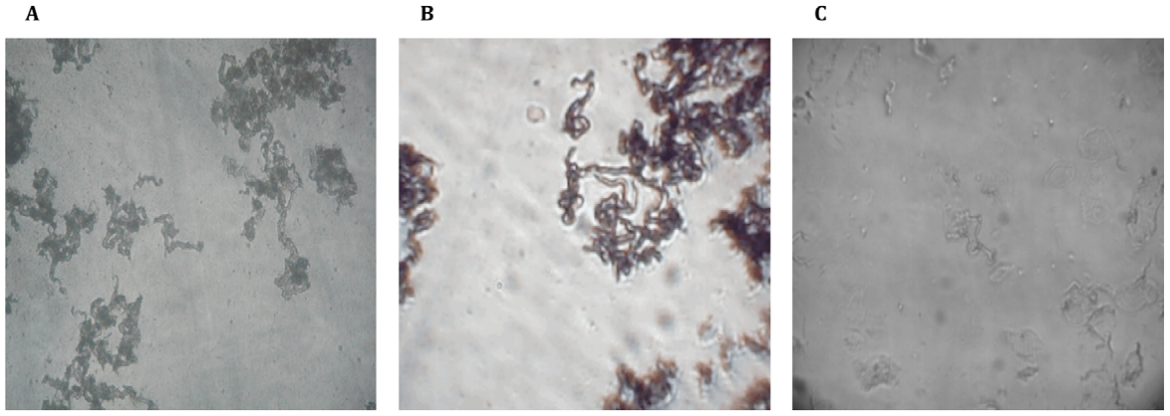
Digital images of *Mycobacterium tuberculosis* MODS cultures. (a) Characteristic image of day 9 cording pattern of MTB in MODS captured with a NIKON Eclipse TS100-F inverted microscope (100X) and a 2 Mpixels Olympus CCD camera1; (b) Characteristic cording image captured by prototype 1 at 100× magnification (day 14); (c) Characteristic cording image captured by prototype 2 at 100× magnification (day 9).

Previously, MODS plates have been visualized only using an inverted light microscope (in our laboratory the NIKON Eclipse TS100-F with an infinity correction optical system). Where digitalization of images has been necessary for electronic transmission and remote diagnosis^8^, photomicrographs have been captured using a CCD (charge-coupled device) camera (Olympus C-3030) with 4 Mpixels resolution attached to the photo port. Typical image of the cording colonies of MTB in MODS taken using a 10X objective (NA of 0.25) and 10X eyepiece is shown in figure 1a.

### Prototype 1

**Figure 2:**
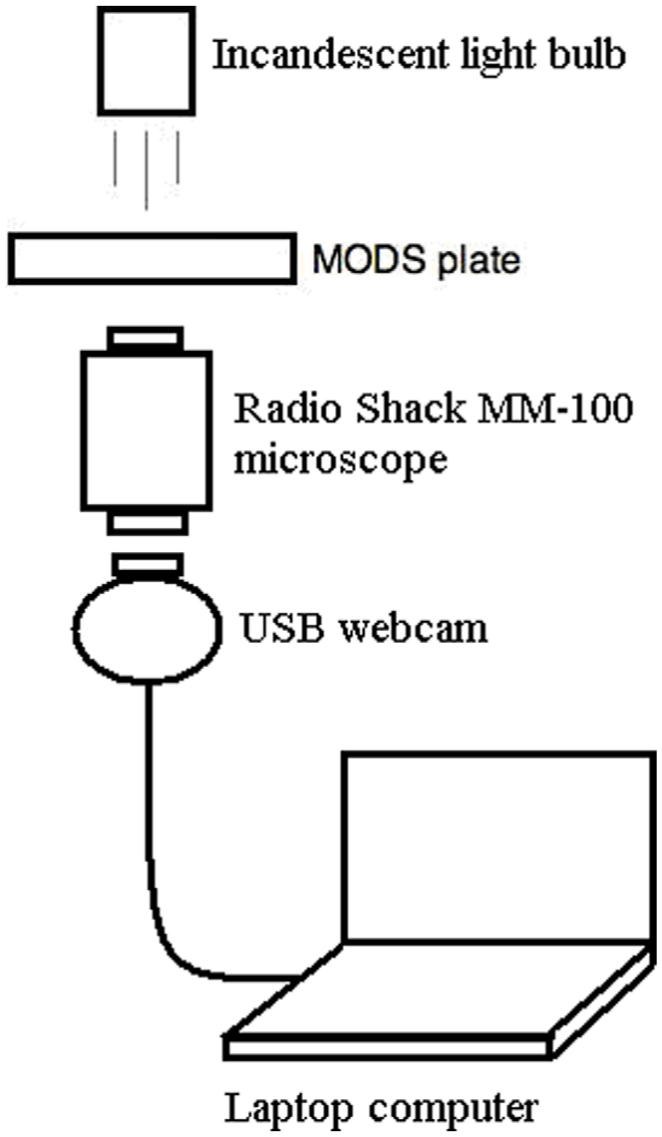
Schematic diagram of the prototype 1 system. Magnifier and digital camera prototype with output to laptop.

The first prototype attempt utilised a simple system composed of a 60–100× magnification pocket microscope (Radioshack MM-100, $10) and a standard USB 320×240 resolution webcam. A 0.5 W standard incandescent light bulb and a simple condenser lens were used to illuminate the sample from above and the captured image was visualized and stored on a laptop computer. The magnification microscope was fixed in an upright position 5 mm beneath the MODS plate. The webcam lenses were removed, and the webcam was attached to the ocular of the magnifier with 15 mm separation between the ocular and the CMOS (Complementary Metal Oxide Semiconductor) sensor of the webcam. The system focus and zoom were adjusted directly through the magnifier controls. The webcam was connected to a laptop through the USB port. A schematic diagram of this prototype is shown in figure 2.

### Prototype 2

The second generation inverted microscope was a stand-alone model based on a 100× magnifier, a modified digital camera with SD/MMC (secure digital/multimedia memory card) memory slot and TV output, and a 5-inch LCD monitor on which to visualize the captured images. The magnifier was composed of a set of standard DIN (Deutsche Industrie Norm) 10X objective (achromatic, NA 0.25) and 10X eyepiece (Huygenian), supported in an aluminium L-shaped optical base. This base was composed of two black painted tubes and a 45° mirror, obtaining a total optical length of 160 mm (as recommended for DIN standard). The MODS plate was located above the objective and focussing was achieved by a mechanical stage. The stage moved the plate support varying its distance to the objective lens. A digital camera (Genius DV600, working at resolution of 2048×1536 pixels) was modified for this prototype. The CMOS sensor was taken out of the camera, and mounted in a cap that could be easily attached to the eyepiece of the magnifier when working in digital mode. The CMOS sensor was removed from the eyepiece when working in standard mode. The eyepiece-sensor separation distance was 20 mm. The combined CMOS-Magnifier system was moved using the mechanical stage. An earthed flat cable with aluminium foil was used to electronically connect the CMOS sensor and the rest of the camera.

**Figure 3:**
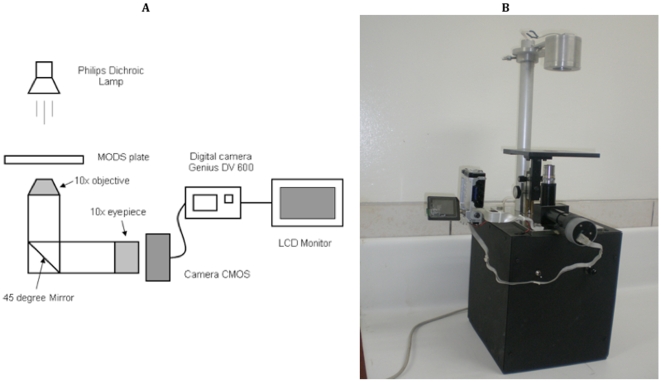
Prototype 2 system. (a) **Schematic diagram of the prototype 2 system.** Magnifier and digital camera prototype with output to digital screen; (b) photograph of prototype 2.

**Figure 4:**
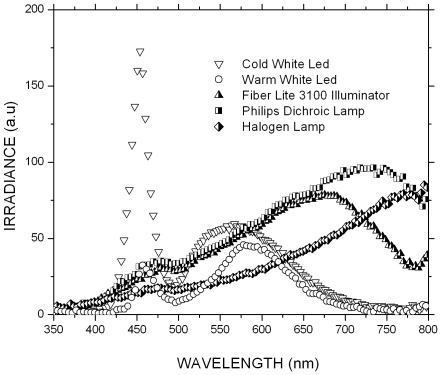
Emission spectra of different illumination systems.

Observed down a microscope the image of the MODS well fills the field of view of the microscopist; by comparison when working in digital mode, the image on the camera LCD screen is tiny. In order to generate images of adequate size an external 5 inch LCD monitor (Denver DFT-507) was used. This enabled the operator to directly inspect the culture on the screen in order to determine whether the characteristic cording growth was present and to focus appropriately before capturing a digital image to be transmitted for remote diagnosis. A schematic diagram and photograph of this prototype are shown in figure 3. Illumination was provided by a 12 V, 50 W Philips halogen dichroic lamp ($2), positioned 170 mm from the plate. Alternate illumination systems were tested, including a regular 20 W halogen bulb, several LEDs and a condenser lens. As a reference illumination system, we tested the commercial high-end halogen lamp (Fiber Lite 3100, Dolan-Jenner Industries, Boxboro, MA). The emission spectra of the different illumination systems were measured with a spectrophotometer (Ocean Optics USB4000, Dunedin, FL). The illumination system that produced the best quality images as compared to the ones observed in the commercial inverted microscope was the 50 W Philips halogen dichroic lamp. This illumination system also showed the most similar emission spectra compared to the Fiber Lite 3100 lamp (figure 4).

This prototype was able to operate in both standard and digital mode. In “standard mode” reading was performed by direct observation through the eyepiece by the laboratory technician. In “digital mode” the reading could be performed locally, by observation of the LCD screen or remotely, by transmission of the captured digital image to a distant site where an expert is available to complete the reading.

The cost for this system was $320, less than one tenth of the cost of the commercial high-end inverted microscope. The image quality achieved is shown in figure 1c.

### Validation

#### Performance of the prototype 2 system for local TB diagnosis in manual mode

In order to determine the performance of the prototype 2 system in manual mode, we compared the readings (MTB present or absent) of 118 nine-day-old MODS cultures using the prototype 2 in standard mode with those using the Nikon microscope. Nine-day old cultures were chosen because this timepoint allows for a fair spread of clear-positive and early positive cultures - 90% of culture-positive samples are detectable in MODS by day 9. Cultures were selected consecutively and anonymously from the routine patient samples from TB suspects being processed in the laboratory; none of the study results were linked to the patient or their standard MODS culture report. All samples were read blindly twice using the Nikon microscope and the prototype 2 system by the same technician within 4 hours of each other. A second technician read all the samples using only the Nikon microscope on the same day. Plates were presented in random order and without identifying features so technicians were unaware of previous reading results.

#### Performance of the prototype 2 system for local TB diagnosis in digital mode

The performance of the prototype 2 system in digital mode was similarly evaluated by the same two technicians. The readings of 153 nine-day-old MODS cultures using observation of the 5″ LCD screen of prototype 2 were compared with those using the Nikon microscope. For logistical reasons, the readings of the two technicians with the Nikon microscope were performed 24 hours apart; the readings conducted by the same technician with the two microscopes were completed within 5 hours.

#### Performance of the prototype 2 system for remote TB diagnosis

To determine the performance of the prototype 2 system for remote TB diagnosis, a total of 30 nine-day-old MODS cultures were read in standard mode and compared to the readings of the digitally captured images. The digital images were captured at the maximum resolution (3 Mp) and transferred to a computer using an SD card reader. The same technician analyzed the images using a regular image visualization software allowing zooming and contrast optimization.

## Results

### Performance of the prototype 1 system

With this low-cost setup (Magnification microscope and Webcam for $25) it was possible to recognize the characteristic MTB cording, demonstrating proof of principle. A MODS image captured by this system is shown in figure 1b.

Though the image-capture device was inexpensive and simple, this system required computer hardware and software; dispensing with the need for a microscope whilst creating the need for a computer did not address the underlying need for simplified technology. Furthermore, because of the limitation of the optical system cords were only clearly appreciated in cultures of 14+ days, somewhat attenuating the marked rapidity of conventional MODS (median 7 days to detection) [3].

### Performance of the prototype 2 system for local TB diagnosis in manual mode

For readings with the Nikon microscope, the inter-technician agreement was 98.31% (Kappa statistic = 0.9661, *P*<0.0001). The agreement between the readings with the prototype 2 and the readings with the Nikon microscope completed by the same technician was 96.61% (Kappa statistic = 0.9322, *P*<0.0001, 49% MODS culture-positive by Nikon reading); the level of agreement between the Nikon microscope and the prototype 2 system in standard mode is high and within the range of the between-technician variability.

### Performance of the prototype 2 system for local TB diagnosis in digital mode

Inter-technician agreement using the Nikon microscope was 93.5% (Kappa statistic = 0.8690, *P*<0.0001). Inter-microscope agreement for readings by the same technician was 94.12% (Kappa statistic = 0.8819, *P*<0.0001, 56% MODS culture-positive by Nikon reading). Since agreement of the prototype with the Nikon microscope was marginally lower for digital (on-screen) reading than for standard (eyepiece) reading, it would be reasonable to consider using the eyepiece for local reading and the digital mode only for when digital image capture and transmission is required.

### Performance of the prototype 2 system for remote TB diagnosis

The comparison of these two readings showed 100% agreement. These results suggest that the prototype 2 could also be used as a platform for TB telediagnosis by digitalizing MODS cultures images for further transmission and analysis in a remote site, as previously proposed^8^.

## Discussion

The success of efforts to roll-out and scale up MODS is likely to depend largely upon the ease with which the methodology can be performed and the cost. In many settings an important potential obstacle could be the need for an inverted light microscope with attendant capital and maintenance costs. Our hypothesis was that the high specification of a commercial inverted light microscope might not be necessary for the relatively simple image requirements of MODS plate reading. These proof-of-principle experiments with easily constructed prototypic inverted microscope replacements confirm this to be the case.

We have clearly demonstrated that MODS plate images of sufficiently high quality may be captured utilising relatively simple and widely available technology at a fraction of the cost of a high-performance inverted light microscope. Factory production of such a tool could yield an even cheaper product; bringing access to high quality TB and MDRTB diagnosis through MODS culture one step closer to many more people living in high-burden, resource-limited settings.

In a previous study we demonstrated the utility of mobile phones to transmit MODS digital images for remote diagnosis [8]. Images were captured by a commercial inverted digital-microscope-system and transmitted to a remote site where pattern recognition was performed by trained personnel. Following the same procedure, the use of mobile phones/internet and a system like the prototype 2 which allows low-cost digital capture of MODS images, could be used as a platform for TB telediagnosis or as a tool for training of laboratory personnel through distance learning, for resolution of equivocal appearances and for remote quality assurance, all of which are particularly important and largely neglected in resource-limited settings. Though we have used MODS as the basis for development of this simplified inverted microscopy platform it could clearly also be adapted for other applications currently otherwise dependent upon expensive branded equipment.
